# Insomnia in Iranian Traditional Medicine

**DOI:** 10.5812/ircmj.15981

**Published:** 2014-03-05

**Authors:** Zohre Feyzabadi, Farhad Jafari, Parvin Sadat Feizabadi, Hassan Ashayeri, Mohammad Mahdi Esfahani, Shapour Badiee Aval

**Affiliations:** 1 Iranian Traditional Medicine Department, Medicine Faculty, Shahed University, Tehran, IR Iran; 2 Health and Social Medicine Department, Medicine Faculty, Shahed University, Tehran, IR Iran; 3 General Linguistics Department, University of Tehran, Tehran, IR Iran; 4 Department of Neurosciences, Rehabilitation Faculty, Iran University of Medical Sciences, Tehran, IR Iran; 5 Quran, Hadith and Medicine Sciences Research Center, Tehran University of Medical Sciences, Tehran, IR Iran; 6 Complementary and Traditional Medicine Faculty, Mashhad University of Medical Sciences, Mashhad, IR Iran

**Keywords:** Sleep Initiation and Maintenance Disorders, Brain, Medicine, Traditional

## Abstract

**Context:**

Insomnia is one of the most prevalent sleep disorders characterized by sleep difficulty that impairs daily functioning and reduces quality of life. The burden of medical, psychiatric, interpersonal, and societal consequences of insomnia expresses the importance of diagnosing and treatment of insomnia. The aim of study was to investigate causes of insomnia from the viewpoint of Iranian traditional medicine.

**Evidence Acquisition:**

In this review study, we searched insomnia in a few of the most famous ancient textbooks of Iranian traditional medicine from different centuries. This books includeThe Canon of Medicine by Avicenna (the first version of Beirut), Zakhire Kharazmshahi by Jurjani (the scanned version of Bonyade Farhang-e Iran), Malfaregh by Razes (the first version of Iran University of Medical Sciences), and Aqili’s cure by Aqili (the first version of Iran University of Medical Sciences).

**Results:**

This study found that in Iranian traditional medicine manuscripts, insomnia was called sahar and even though many factors induce insomnia, most of them act through causing brain dystemperament.

**Conclusions:**

The brain dystemperament is considered one of the main causes of insomnia and insomnia can be well managed with an organized line of treatment, by correcting the brain dystemperament through elimination of causes. This study helps to find new solutions to treat insomnia.

## 1. Context

Sleep is a normal condition of the body which occurs periodically and is associated with a depression of physiological function (1). The term insomnia is used in a variety of ways in medical literature and popular press. Most often, it is defined by the presence of an individual’s report of difficulty with sleep, such as difficulty in sleeping, difficulty in falling sleep, or staying sleep (2). It is never defined based on the number of hours because the amount of sleep obtained during the night varies among individuals. The reason is that many factors affect daily sleep need. Genetic factors, age, and medical or psychiatric disorders are among the factors that strongly influence sleep pattern. For example, sleep requirement may decrease with age (3).

Many studies have shown insomnia as one of the most common sleep disorders all over the world. Although some studies indicated its different prevalence in different countries (2, 4-6). It has been shown that almost 30 percent of people in each society report insomnia in a period of their life, among which 10 percent suffer from chronic insomnia (6). Demographic factors (e.g. aging, female gender, and living alone) are amongst the most commonly hypothesized predisposing factors of insomnia (7). Insomnia affects many aspects of patient’s life, as well. Fatigue, day time drowsiness, reduced memory and concentration, and work disturbance are amongst the adverse effects of insomnia (4).

To treat insomnia, Benzodiazepines are frequently prescribed but they increase the probability of adverse effect such as amnesia (6), slowness, sleepiness, nervousness, forgetfulness, irritability, dizziness, and confusion (8). The complaints of insomniacs about insomnia itself and its treatments’ side effects highlight the importance of reviewing insomnia diagnosis and treatments in complementary medicine. In this writing, we discussed insomnia etiology and its signs and symptoms from the viewpoint of some of the most famous Iranian traditional physicians such as, Razes (865 – 935A.D.), Avicenna (980-1037A.D.), Jurjani (1042-1136 A.D.), and Aqili (18th A.D.).

Iranian traditional medicine, hereinafter called ITM, is an ancient temperamental medicine with a rich literature about insomnia. In ITM, temperament meansthe dominant quality of the composite object and it is made of the interaction of four basic elements (hot, cold, wet, and dry) (9). Temperament has an important function in maintaining the ideal healthy state of an individual. Vulnerability of temperament to alteration, which is called dystemperament, leads to several different types of diseases (10) such as insomnia.

In ITM, the principle of treatment of any disease is elimination of its major causes and modifying the six principles of healthy life (11). These principles are air, food and drink, sleep and wakefulness, body movement and stillness, retention and evacuation, and perturbations of mind (12, 13). In this viewpoint, normal slumber and wakefulness are essential for health. Sleep is an ideal form of rest, physically as well as mentally. Lack of sleep causes dissipation of energies, mental weakness, and digestive disturbances (14). This study aims to investigate insomnia causes, signs, and symptoms in ITM manuscripts to find a new avenue towards curing it. Diagnosis of clinical signs and symptoms of insomnia and recognition of brain dystemperament, which causes insomnia, can be used as a complementary and effective treatment to improve patient’s brain temperament and consequently cure insomnia.

## 2. Evidence Acquisition

### 2.1. Methodology

In this review study, we studied printed editions of four most important ITM books, namely The Canon of Medicine by “Avicenna” (10th and 11th centuries), Zakhire Kharazmshahi by “Jurjani” (11th and 12th centuries), Aqili’s Cure by “Aqili” (18th century), and Malfaregh by “Razes” (9th and 10th centuries). The first three books are among the most important clinical texts and have widely been taught in traditional medicine schools in Iran. The last book is the only book in differential diagnosis of diseases in ITM. As insomnia is called sahar and it is considered as one of the head and brain diseases; we first studied chapters on head and brain diseases, and then searched the exact term of insomnia (sahar) in the books. Then, we collected issues about insomnia definition, etiology, and clinical features and classified the result based on the causes, symptom, and signs. To further understand the pathophysiology of insomnia, sleep physiology was discussed briefly using viewpoints in The Canon of Medicine and Zakhire Kharazmshahi.

### 2.2. Sleep Physiology in ITM

Sleep occurs when the heat moves towards the body inside, affects food moisture and evaporates it. This vapor reaches the brain and relaxes neural tracts or compresses them on each other. In this perspective, the main cause of sleep is moisture. Thus, those whose bodies have more moisture need more sleep. In contrast, people with dry temperament require less sleep than those with wet temperament, and therefore, are more vulnerable to insomnia (14).

### 2.3. Moisture in ITM

Avicenna believes that there are two kinds of natural moisture in the body.

a) Primary moisture (quadruplet humors); after digestion, food is converted to a fluid called humor. The humor can be classified into four types: blood, phlegm, yellow bile, and black bile. All these four classes are called primary moisture (15) and the foundation of health is the right ratio and specific balance of humors based on their quality and quantity (16). b) Secondary moisture; waste moisture which must be excreted from the body, for instance, sweat (15).

### 2.4. Sleep and Wake Definition in ITM

To define sleep and wake, it is necessary to understand the meaning of spirit. Spirit in this article is equivalent to “essence”. However, in ITM it is known as “spirit” (tenuous steamy matter formed from tenuous particles of humors) and heart is introduced as the source of spirit (17, 18). The spirit flows in the body through the blood in the arteries and carries three faculties: natural, vital, and psychic (17).

ITM defines sleep as tendency of psychic faculty and as a result, instinctive heat to move towards the body inside to rest powers and digest the food. According to this definition, the sleeping process makes the body surface cold and causes needing a blanket during sleep (14, 19). In contrast, wake is defined as psychic faculty’s tendency to move towards body outside and stimulating all body organs and make them do what they are capable of. That is why traditional physicians know sleep as being static and wake as moving.

### 2.5. Normal Sleep Characteristics

In ITM, normal sleep must have these features:

1. Occurring after completion of digestion process in stomach. Otherwise, it causes ill-digestion and produces bloat.
2. Being deep.
3. Being continuous.
4. Having normal duration.
5. Not with supine body position. The best position to start sleep is on the right side, and then turn on the left side (15).
6. Not happening during the daylight. “Aqili” expressed three reasons for importance of night sleep: (a) Getting used to sleeping during night. (b) Cold weather at night makes heat tend to move towards the body inside, complete digestion, and create moisture that is the main material of sleep. (c) Night pacifies the senses due to darkness, while daylight makes senses move and does not let the body relax. According to this viewpoint, deep and good sleep does not occur during the daylight (20).
7. Not sleeping under moonlight or sunlight.
8. Using a blanket due to heat transmission towards body inside which makes the body surface cold (14, 15).

### 2.6. Insomnia Definition in ITM

Insomnia is called sahar, which lexically means wake. however, according to “Avicenna” (15), “Razes” (21), “Aqili” (20), and “Jurjani” (14) the excessive wake, which is out of normal range, is known as sahar (hereafter, called insomnia).

### 2.7. Insomnia Etiology in ITM

ITM physicians expressed causes of insomnia as follows:

1. Physical disorders. Any physical problem that causes brain dystemperament can cause insomnia. Different kinds of brain dystemperament and conditions that cause brain dystemperament are explained here:
  1. Simple dry dystemperament of the brain. In this case, insomnia occurs due to dominance of dry temperament in the brain (14, 15, 20, 21); because the natural temperament of the brain is cold and wet. “Razes” suggests that pain, hunger, depression, noise, ill-digestion, and physical weakness induce dryness in the brain and therefore insomnia occurs (21).
  2. Simple warm and dry dystemperament of the brain. In this case, due to dominance of heat along with dryness, the intensity of insomnia is more than the first case because the heat dominance causes the psychic faculty to move towards the body outside and consequently induces insomnia (14, 15, 20).
  3. Bile humor dominance in the body. In this case, bile humor dominance causes dryness and heat in the brain (20) due to the warm and dry nature of bile humor (22).
  4. Black bile humor dominance in the body. In this case, due to black bile humor and fright dominance, the psychic faculty tends towards the body outside and causes insomnia (20, 21) because of the cold and dry nature of black bile (22).
  5. Buriqi or salty moisture of the brain (14, 15, 20). “Aqili” defines buriqi moisture as “a kind of non-natural moisture which is highly affected, burned, and incinerated by heat”. Due to being torrid, this moisture irritates the brain and causes brain dystemperament. This dystemperament usually happens in elders. This adventitious moisture in elders’ body causes them to nap all the time, but not having deep and pleasant sleep. The reason is that even though there is a kind of moisture in the brain (buriqi moisture) which causes sleep, the moisture exists along with the heat that causes awaking frequently (20).
2. Psychological disorders. For example, suffering from depression, fear, or distressing thoughts (20, 21). in addition, “Jurjani” believes that happiness can lead to insomnia, as well (14).
3. Fever. Fever moves torrid and warm vapors towards the brain and leads to insomnia (14, 15, 20, 21).
4. Ill-digestion (14, 20, 21). Excessive eating at night makes bothersome postprandial fullness and ill-digestion and thus, produces bloat. This makes pain and extension feelings in stomach and awakes the person and therefore, lead to interrupted sleep. However, when the person is awake, the undigested food and the produced bloat can be expelled by vomiting and burping, respectively (20).
5. Excessive congestion of body due to bad humors (14, 15, 20).
6. Pain (14, 20, 21). Pain prohibits body organs from their natural activities and makes them resist against aching and try to overcome the problem and therefore, prevents sleep (20).
7. Upward movement of rancid vapors towards the brain due to eating flatulent and vaporous food. Beans (14, 15, 20), lentil (14, 20), leek, and fenugreek (20) causes upward movement of vapor towards the head, heavy headed feeling, headache, depraved elusion, nightmares, and consequently awaking at night and fearing during sleep.
8. Brain cancer (14, 15).
9. Old age. Insomnia occurs more commonly in elderly. The reason is brain dry temperament and salty moisture in elders’ body (14).
10. Sarsam is hot inflammation of the brain that causes insomnia (21)
11. Shift work (20)
12. Eating less than enough which leads to dry temperament (20)
13. Brightness at sleep location (15)

Table 1 summarizes the ITM viewpoints in different centuries about etiology of insomnia.

**Table 1.** Viewpoints of Iranian Traditional Physicians About Insomnia Etiology

Scientist Name	Century or Year (AD)	Etiology of Insomnia
**Razes**	865-935	Simple dry dystemperament of the brain which appears due to hunger, ill-digestion, Pain, depression, and physical weakness
		Fever and sarsam
		Black bile humor dominance (21)
**Avicenna **	980-1037	Simple dry dystemperament of the brain, simple warm and dry dystemperament of the brain, brain salty moisture, pain, brightness at sleep location, distressing thoughts, ill-digestion, excessive congestion of body due to bad humor, flatulent foods e.g. bean, fever, and brain cancer (15)
**Jurjani **	1042-1136	Simple dry dystemperament of the brain, warm and dry dystemperament of the brain, brain salty moisture, pain, depression, happiness, ill-digestion, excessive congestion of body due to bad humor, flatulent foods e.g. bean and lentil, fever, brain cancer, old age (14)
**Aqili**	18	Simple dry dystemperament of the brain, simple warm and dry dystemperament of the brain, bile or black bile humor dominance, brain salty moisture, fever, pain, ill-digestion, excessive congestion of body due to bad humor, depression, fear, distressing thoughts, shift work, overeating, not enough eating, flatulent food e.g. bean, lentil, leek, and fenugreek (20)

### 2.8. Insomnia Clinical Signs and Symptoms

The first step towards understanding etiology of insomnia is recognizing its signs and symptoms. This subject was explained in details by Iranian traditional physicians based on the kind of brain dystemperament. ITM scholars believe that each person has a general temperament, which is determined by the humor dominance in the body, and an organic temperament, which is specific for each body organ (23); for example, natural brain temperament is cold and wet. As brain dystemperament causes insomnia, anything that disturbs brain normal temperament can be considered as insomnia etiology. Herein, we explain clinical signs and symptoms that help diagnosing the kind of brain dystemperament, which induces insomnia:

1. Simple dry dystemperament of the brain. If dominance of solely dryness exists in the brain, the signs will be dryness of eyes, tongue, and nose and head lightness (14, 15, 20, 21).

2. Simple dry and warm dystemperament of the brain. In this case, there are some heat dominance signs, such as extreme thirst, feeling irritation and heat in the head and eyes, and also some signs of brain dryness (such as what was mentioned in 1). In addition if you touch the head of the person, it is felt warm (14, 15, 20).

3. Bile humor dominance in the body. In this case, bile dominance signs and signs of brain dryness such as bitterness of mouth, yellow face and eyes, dryness of nose, decrease of appetite, and vomiting appear (20).

4. Black bile humor dominance in the body In this case, black bile dominance signs such as fear during sleep, nightmares, and awaking at night with fear appear and the person feels heavy headed (20).

5. Aggregation of buriqi moisture in the brain. In this case, as some moisture moves from brain towards eyes and nose, nasal and eye discharge are seen. In addition, due to buriqi moisture dominance, the person feels heavy headed. If these signs happen in elderly, it is assumed that there is buriqi moisture in the brain (14, 15, 20).

### 2.9. Complications of Insomnia

Extreme wake leads to energy consumption, hunger, and increase appetite and if reaches the limit to be called insomnia, digestion is not performed completely. In other words, digestion process needs heat and moist while wakefulness moves heat towards the body outside (15), hence, there is not enough heat in the body to perform the digestion process, which leads to incomplete digestion (24). Consequently, it causes incomplete nutrition supply for body organs and finally dryness and slimness of the body (15).

## 3. Discussion

ITM assumes that sleep occurs when vapor reaches the brain and relaxes neural tracts. In this perspective, the main cause of sleep is moisture and natural brain temperament is cold and wet. Thus, anything that makes the brain away from its main nature induces brain dystemperament. Hence, people with brain dry temperament need less sleep and are more vulnerable to insomnia (20). This perspective can be compared with modern medicine that shows different people need different amount of sleep (3). In ITM, insomnia is a temperamental disease that occurs due to brain dryness and most frequent causes of insomnia such as shift work, major depression, distressing thoughts, pain, hunger, depression, noise, ill-digestion, and physical weakness lead to dryness of brain and consequently, they induce insomnia.

Although temperament is not defined in modern medicine, lack of sleep hygiene, medical and psychiatric disease (e.g. depression, anxiety), environmental factors (25), old age (2) comorbid disorder such as pulmonary, cardiac, neurologic, and endocrine diseases, pain disorder (26), and gastroesophageal reflux disease (27), which are considered as insomnia causes in modern medicine, are similar to etiologies of insomnia in ITM. These common points might be signs of deep insight of the ancient physicians and therefore, follow-up treatment would be useful in this field. This paper might help us to recognize causes of insomnia and its predisposing factors from ITM viewpoint to provide a new and applicable classification of insomnia reasons; in fact, ITM is a holistic medicine in which etiology of the disease is very important and the treatment is based solely on removal of the cause and modification of lifestyle.

In our future works, we are going to review insomnia prevention and curing methods in ITM based on the obtained classification. We need to mention some limitations that we faced during this study. ITM is combined by different medical traditions from Greece, Egypt, India, and China from more than 4000 years ago (28). It is a temperamental medicine with specific structure different from other schools and therefore, is not completely comparable to other traditional medicine schools. Consequently, reviews and articles on other traditional medicines were not very helpful. In addition, due to lack of enough papers and review articles on ITM viewpoints, we studied insomnia etiology based on original sources and articles used in this study were often used to explain the specific terms of ITM. In addition, since accessing the original books was not as easy as articles, it was not possible to review all traditional manuscripts on the topic. Furthermore, humoral medicine is a very complex issue and we limited the scope of this study to a brief summary of the main issues involved. However, we hope that this study stimulate some readers to look with a different angle at this issue.
